# Association between serum uric acid and all-cause and cardiovascular-related mortality in hemodialysis patients

**DOI:** 10.3389/fnut.2024.1499438

**Published:** 2024-12-02

**Authors:** Wenyuan Gan, Fan Zhu, Xun Fang, Wenzhe Wang, Danni Shao, Huihui Mao, Wei Xiao, Wenli Chen, Fang Xu, Xingruo Zeng

**Affiliations:** Department of Nephrology, The Central Hospital of Wuhan, Tongji Medical College, Huazhong University of Science and Technology, Wuhan, China

**Keywords:** uric acid, hemodialysis, mortality, cardiovascular-related death, GNRI (geriatric nutritional risk index)

## Abstract

**Background:**

The association between serum uric acid (UA) and all-cause and cardiovascular-related mortality in hemodialysis (HD) patients is conflicting. We investigated this association and explored the effect modification of underlying nutritional status, as reflected in the lean tissue index (LTI) and the Geriatric Nutritional Risk Index (GNRI), which serve as markers of muscle mass and nutritional risk in HD patients.

**Methods:**

A retrospective cohort study was conducted from January 2019 to December 2023. We investigated the association between serum UA and the outcomes using the Cox proportional hazards regression and restricted cubic splines. Subgroup analyses based on the LTI and GNRI were conducted to explore possible effect modification.

**Results:**

During a mean follow-up of 32.9 months, 876 patients who underwent HD were included in the analysis. The association between serum UA and all-cause mortality showed a non-linear U-shaped pattern (*p* = 0.007), with a survival benefit observed for the patients with serum UA levels between 3.4 and 6.8 mg/dL. In the multivariable-adjusted model, the low and high UA groups were associated with a greater risk of all-cause mortality compared to the reference UA group (hazard ratio (HR) =1.24, confidence interval (CI) 1.03–2.12, *p* = 0.027; HR = 1.09; CI 1.05–2.08. *p* = 0.012). In the low UA group, a greater risk of mortality was observed in patients with low LTI (<12.3; HR 1.56, 95% CI 1.22–1.82) and GNRI values (<102.1; HR 1.43, 95% CI 1.12–1.76), but not in those with high LTI and GNRI values. There was no significant association between serum UA and cardiovascular disease-related mortality.

**Conclusion:**

Our study showed that lower and higher serum UA levels increase the risk of all-cause mortality in HD patients. Among the patients with lower UA levels, low LTI and GNRI values showed a greater risk of mortality. This finding suggested that better nutritional status, rather than elevated UA levels, is likely to improve long-term survival in HD patients.

## Introduction

Uric acid (UA) is the end product of purine metabolism and is excreted largely by the kidneys (1). Elevated serum UA levels are the result of impaired renal function, and 40–80% of patients with end-stage kidney disease experience hyperuricemia (1). Even in patients who undergo hemodialysis (HD), the mean serum UA removal is approximately 1 g per HD session (2). Consequently, hyperuricemia remains common in patients who undergo HD (3). In the general population, hyperuricemia has been shown to be associated with an increased risk of gout, hypertension, atherosclerosis, and cardiovascular disease (CVD) by causing endothelial dysfunction, activation of the renin-angiotensin-aldosterone system, and oxidative stress, thereby contributing to vascular smooth muscle cell growth and arterial function impairment (1, 4–11). In addition, UA is known as a potent radical scavenger and antioxidant in the human body. In this context, UA has been shown to prevent the oxidative inactivation of endothelial enzymes and preserve the ability of the endothelium to mediate vascular dilatation during oxidative stress (2). Therefore, it has been suggested that elevated UA levels may be a result of the body’s compensatory mechanism to counteract oxidative damage (12), while lower serum UA levels may reflect inadequate protection against oxidant-mediated stress (13). Interestingly, recent studies have also suggested that UA may serve as a marker of nutritional status in patients undergoing HD (14–16). Protein-rich diets tend to contain large quantities of purines, and higher serum UA levels may represent better nutritional status, which is important for long-term survival in patients who undergo HD. Therefore, there are conflicting data on the association between UA and long-term survival in patients who undergo HD (17–19), with studies reporting direct, inverse, or varying forms of associations (3, 14, 16, 17, 20–25). A better understanding of the reasons behind these conflicting results is necessary to determine the optimal serum UA levels for HD patients. In this study, we examined the association between UA levels and all-cause mortality, as well as cardiovascular disease-related mortality, in patients who underwent HD and explored whether this association was affected by nutritional status.

## Methods

### Study design and study population

We conducted a retrospective cohort study that included data from 876 incident HD patients at the Wuhan Central Hospital dialysis center, the largest dialysis center in Central China, between 1 January 2019 and 31 December 2023. All patients underwent regular dialysis three times a week at a blood flow rate of 250–300 mL/min and a dialysis solution flow rate of 500 mL/min, and the treatments were performed using high-flux biocompatible dialyzer membranes. The following inclusion criteria were applied: (1) Patients aged ≥18 years who underwent HD for more than 3 months and (2) availability of at least one serum UA measurement and body composition monitor examination after 3 months of HD initiation. The exclusion criteria were as follows: (1) Secondary nephropathy caused by autoimmune diseases, hepatitis, malignancy, drugs, and other systemic diseases; patients were excluded if any of the above diagnoses were recorded at any time during the course of the disease; (2) patients who ever underwent or are currently undergoing peritoneal dialysis; (3) patients with kidney transplant; and (4) patients with acute kidney injury. The study was approved by the Medical Ethics Committee of the Wuhan Central Hospital (approval number: WHZXKYL2022-112-01), and the requirement for informed consent was waived due to the retrospective nature of the study.

### Exposure definition and outcome assessment

For each patient, the first measurement of serum UA after 3 months of HD initiation was defined as the exposure and the date of this measurement was considered the index date. The primary outcome of interest was the time to all-cause mortality, while the secondary outcome was the time to CVD-related mortality. CVD-related mortality was defined as death due to acute myocardial infarction, atherosclerotic heart disease, valvular heart disease, cardiomyopathy, cardiac arrhythmia, unspecified cardiac arrest, congestive heart failure, cerebrovascular accident (including intracranial hemorrhage), ischemic brain damage, anoxic encephalopathy, and peripheral vascular disease. If patients died in a hospital, the death certificates were reviewed to determine the exact cause of death. If the death occurred outside of a hospital, the experts would reach a consensus on the cause of death after carefully considering the descriptions provided by the family members. All patients were followed up from the index date until one of the following events occurred: death, kidney transplantation, transfer to peritoneal dialysis, change of dialysis center, loss to follow-up, or the end of the follow-up, whichever took place first.

### Clinical variables

We collected medical information including demographics, comorbidities, body composition, laboratory data, medication, and clinical outcomes. The laboratory parameters and blood pressure were measured at the index date, before the dialysis session. The history of comorbidities was obtained from the medical records. Dialysis vintage was calculated as the time from the initiation of HD to the index date. The body mass index (BMI) and lean tissue index (LTI) were calculated based on the post-dialysis body weight and determined using the body composition monitor, which applies the bioimpedance spectroscopy technique. The LTI used here serve as a marker for muscle mass and reflect protein intake. The Geriatric Nutritional Risk Index (GNRI), which is calculated from a patient’s serum albumin, weight, and height, was used as a simple and accurate tool for predicting the risk of morbidity and mortality (26). The use of xanthine oxidoreductase inhibitors (XORi), including allopurinol and febuxostat, was recorded, as these inhibitors may limit the production of oxidative radicals in patients with end-stage kidney disease, potentially affecting the prognosis (27, 28). The use of these medications was defined as having at least one prescription for the drugs during the follow-up.

### Statistical analysis

The *t*-test and Mann–Whitney U-test was performed to compare normally and non-normally distributed continuous variables. The chi-squared test or Fisher’s exact test was performed to compare categorical variables, as appropriate. The correlations between serum UA levels and other variables were assessed using the multivariable linear regression model. The patients were stratified into three groups based on the serum UA levels: low UA (<5.0 mg/dL), reference UA (5–7 mg/dL), and high UA (>7.0 mg/dL). A Kaplan–Meier analysis was conducted to illustrate the differences in survival between the different UA groups, and comparisons were made using the log-rank test. The Cox proportional hazards regression, evaluating serum UA as a categorical variable (low, reference, and high UA groups), was performed to investigate the unadjusted and multivariable-adjusted associations between serum UA and the outcomes. Variables with a *p*-value of <0.1 in the univariate analysis and the potential biological variables were included in the multivariate analysis (29, 30). To address potential multicollinearity among the variables in our model, we assessed the variance inflation factor for each variable. All values were below 5, indicating that multicollinearity was unlikely to be a significant issue. We conducted the proportional hazards assumption test based on Schoenfeld residuals, which yielded a *p*-value of >0.05. The serum UA levels were also modeled as a continuous variable (per 1 mg/dL increase) to evaluate the possibly non-linear association between serum UA and the outcomes using restricted cubic splines with five knots (31). Non-linearity was assessed using the likelihood ratio test (32). Subgroup analyses were performed to test for effect modification stratified by age, sex, the LTI, and the GNRI. A sensitivity analysis was performed to assess the robustness of the findings using the mean serum UA levels of the patients, with at least three measurements recorded every 12 months. Missing data in our study were less than 3%, and a multiple imputation method was carried out to impute the missing data, assuming that the data were missing at random. The *p*-values were two-tailed, and a p-value of <0.05 was considered statistically significant. Statistical analysis was performed using R version 4.1.1 (R Foundation for Statistical Computing, Vienna, Austria).

## Results

**Figure 1:**
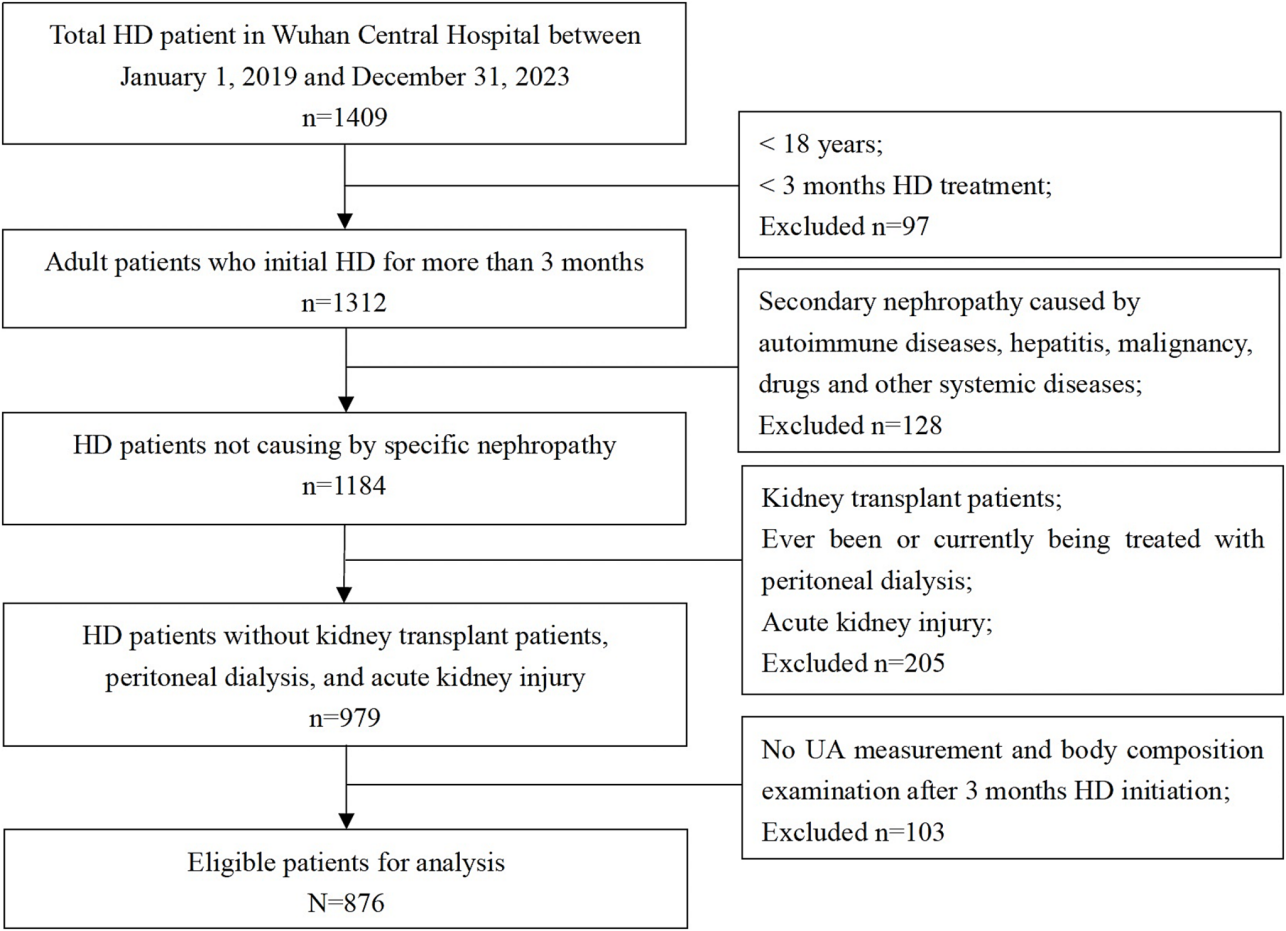
Flowchart of participant selection. Among 1,409 participants from the Wuhan Central Hospital dialysis center, between 1st January 2019 and 31st December 2023, 876 patients were eligible for the analysis.

During a mean follow-up of 32.9 ± 12.2 months, 876 patients with a median age of 63.6 ± 13.1 years were included in this study. A total of 162 (18.5%) all-cause deaths and 71 (8.1%) CVD-related deaths were recorded during the follow-up period. A flowchart displaying patient selection is presented in Figure 1. Baseline characteristics on the index date are presented in Table 1 for the overall cohort and different serum UA level groups (low [<5.0 mg/dL], reference [5.0 to 7.0 mg/dL], and high [>7.0 mg/dL]). The mean serum UA level of all patients was 6.4 ± 1.4 mg/dL, and the percentages of the patients in the low, reference, and high groups were 27.6, 48.7, and 23.6%, respectively. Of all patients, 132 (15.1%) received XORi.

**Table 1:** Baseline characteristics of the overall cohort and the different UA level groups at the index date.

Characteristics	Different UA groups				
	Total	Low (<5.0 mg/dL)	Reference (5.0–7.0 mg/dL)	High (>7.0 mg/dL)	*p*-value
No. of patients	876	242	427	207	<0.001
Age (years)	63.6 ± 13.1	66.4 ± 12.9	62.5 ± 14.1	56.6 ± 11.4	<0.001
Male (%)	508 (58.0%)	124 (51.6%)	253 (59.3%)	131 (62.8%)	<0.001
BMI (kg/m^2^)	26.3 ± 6.3	25.9 ± 6.2	26.5 ± 6.6	27.4 ± 6.0	<0.001
LTI (kg/m^2^)	12.3 ± 2.8	11.5 ± 3.1	12.6 ± 2.9	13.1 ± 2.7	<0.001
GNRI	102.1 ± 14.2	96.3 ± 15.2	105.2 ± 13.5	109.6 ± 14.7	<0.001
Hypertension (%)	775 (88.5%)	215 (88.8%)	375 (87.8%)	185 (89.3%)	0.335
Diabetes mellitus (%)	383 (43.7%)	116 (47.9%)	187 (43.8%)	80 (38.6%)	<0.001
CVD (%)	261 (29.8%)	73 (29.8%)	124 (29.0%)	64 (30.9%)	0.193
SBP (mmHg)	140.4 ± 18.5	141.6 ± 20.2	138.6 ± 18.4	140.9 ± 20.3	0.554
DBP (mmHg)	76.6 ± 14.6	77.3 ± 14.1	75.6 ± 11.8	76.9 ± 15.3	0.671
Hemoglobin (g/dl)	9.7 ± 2.0	9.4 ± 1.9	9.8 ± 2.0	9.9 ± 2.1	<0.001
Albumin (g/dl)	3.9 ± 3.1	3.7 ± 3.4	3.9 ± 3.5	4.2 ± 3.3	<0.001
Calcium (mmol/L)	2.2 ± 0.2	2.2 ± 0.2	2.2 ± 0.2	2.1 ± 0.2	0.906
Phosphorus (mmol/L)	1.6 ± 0.5	1.3 ± 0.4	1.6 ± 0.6	1.9 ± 0.5	<0.001
Potassium (mmol/L)	5.2 ± 0.7	5.1 ± 0.7	5.3 ± 0.8	5.1 ± 0.7	0.759
Parathyroid hormone (pmol/L)	280.3 ± 200.1	244.1 ± 190.1	270.1 ± 233.9	337.8 ± 227.6	<0.001
C-reactive protein (mg/L)	4.0 ± 1.8	3.9 ± 1.8	4.0 ± 2.5	3.9 ± 2.0	0.573
Ferritin (ug/L)	133.5 ± 115.6	136.9 ± 117.6	130.2 ± 113.3	134.5 ± 110.2	0.417
UA (mg/dL)	6.4 ± 1.4	4.2 ± 1.1	6.1 ± 0.9	7.9 ± 1.5	<0.001
Total cholesterol (mmol/L)	3.8 ± 1.3	3.6 ± 1.1	3.8 ± 1.0	4.1 ± 1.1	<0.001
Triglyceride (mmol/L)	1.5 ± 1.3	1.3 ± 0.9	1.5 ± 1.4	1.7 ± 1.0	<0.001
Serum creatinine (mg/dL)	7.3 ± 2.6	5.8 ± 2.7	6.9 ± 2.6	8.7 ± 2.9	<0.001
XORi	132 (15.1%)	42 (17.4%)	69 (16.2%)	21 (10.1%)	<0.001
Dialysis vintage (months)	3.1 ± 1.7	3.1 ± 1.5	2.9 ± 2.0	3.2 ± 1.7	0.775
Follow-up (months)	32.9 ± 12.2	32.0 ± 13.5	32.5 ± 11.5	33.4 ± 12.6	0.654

UA, uric acid; different UA groups, low (<5.0 mg/dL), reference (5–7 mg/dL), and high (>7.0 mg/dL) UA groups; BMI, body mass index; LTI, lean tissue index; GNRI, Geriatric Nutritional Risk Index; CVD, cardiovascular disease; SBP, systolic blood pressure; DPB, diastolic blood pressure; and XORi, xanthine oxidoreductase inhibitors.

Compared to patients in the low UA group, the high UA group patients were significantly younger (56.6 ± 11.4 vs. 66.4 ± 12.9 years); had higher BMI (27.4 ± 6.0 vs. 25.9 ± 6.2), LTI (13.1 ± 2.7 vs. 11.5 ± 3.1), and GNRI (109.6 ± 14.7 vs. 96.3 ± 15.2) scores; had higher serum levels of albumin (4.2 ± 3.3 vs. 3.7 ± 3.4 g/dL), phosphorus (1.9 ± 0.5 vs. 1.3 ± 0.4 mmol/L), creatinine (8.7 ± 2.9 vs. 5.8 ± 2.7 mg/dL), total cholesterol (4.1 ± 1.1 vs. 3.6 ± 1.1 mmol/L), and triglycerides (1.7 ± 1.0 vs. 1.3 ± 0.9 mmol/L); were mostly male (62.8% vs. 51.6%); had a lower prevalence of diabetes (38.6% vs. 47.9%); and were using urate-lowering agents (13.5% vs. 20.2%). For all these comparisons, the *p*-value was <0.001. The prevalence of hypertension and CVD did not differ between the various UA groups. After adjusting for demographics, comorbidities, and laboratory variables, the multivariable linear regression model showed that the serum UA levels were positively correlated with the LTI (*r* = 0.17; *p* < 0.001) and GNRI (*r* = 0.21; *p* = 0.004) scores, which serve as proxy indicators of nutritional status.

**Figure 2:**
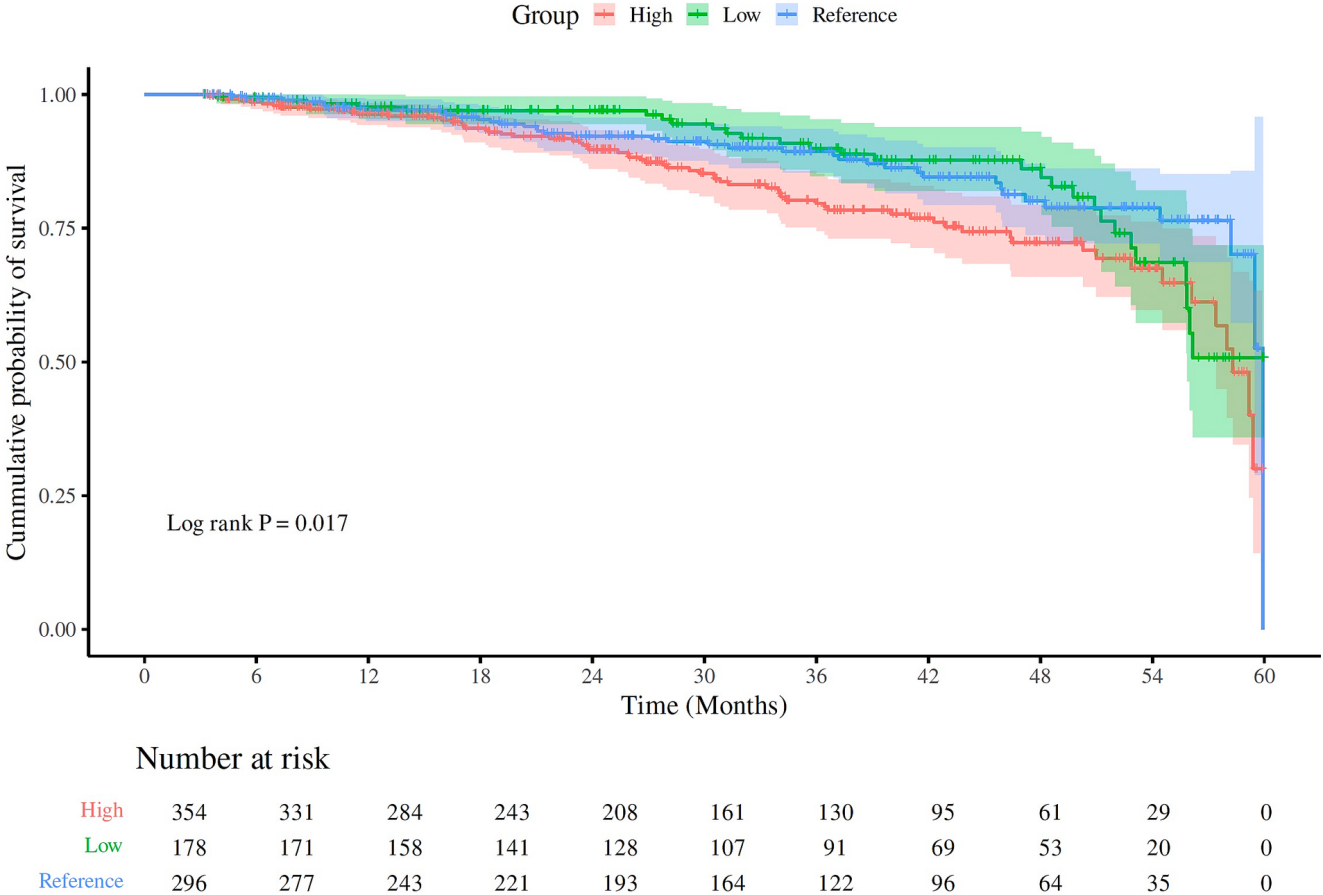
The Kaplan–Meier curves for all-cause mortality in the different UA groups. The different UA groups were classified as low (<5.0 mg/dL), reference (5–7 mg/dL), and high (>7.0 mg/dL). The patients in the high UA group, followed by the patients in the low UA group, had higher all-cause mortality compared to the patients in the reference UA group (log-rank χ^2^ = 4.8; *p* = 0.017).

The Kaplan–Meier survival curves confirmed that the patients in the high UA group and low UA group had a greater risk of all-cause mortality compared to those in the reference UA group (log-rank test/chi-square = 4.8; *p* = 0.017), as shown in Figure 2. However, the risk of CVD-related mortality was not statistically different among the different UA groups (log-rank χ^2^ = 0.72, *p* = 0.40).

The Cox proportional hazards model was used to quantify the associations between the different UA groups and all-cause and CVD-related mortality. Table 2 shows the unadjusted and multivariable-adjusted hazard ratios (HRs) with 95% confidence intervals (CIs) based on serum UA levels as a categorical variable. After adjusting for age, sex, dialysis vintage, comorbidities, BMI, LTI, GNRI, hemoglobin, albumin, phosphorus, parathyroid hormone, total cholesterol, triglyceride, creatinine, and UA lowering agents, the low and high UA groups were both associated with a greater risk of mortality compared to the reference UA group (HR = 1.24, CI 1.03–2.12, *p* = 0.027; HR = 1.09; CI 1.05–2.08. *p* = 0.012). Although the risk of mortality was found to be increased in both the low and high UA groups, it was notably higher in the low UA group (HR = 1.24) compared to the high UA group (HR = 1.09). The low and high UA groups were also associated with a greater risk of CVD-related mortality, but this association did not reach statistical significance.

**Table 2:** Associations between the different UA groups and all-cause and CVD-related mortality.

Characteristics	HR (95% CI) and P-value			
	Unadjusted	*P*-value	Multivariable-adjusted	*P*-value
All-Cause Mortality				
Low UA group	1.42 (1.08–2.47)	0.012	1.24 (1.03–2.12)	0.027
High UA group	1.25 (1.06–2.51)	0.017	1.09 (1.05–2.08)	0.012
CVD-related Mortality				
Low UA group	1.36 (0.97–2.98)	0.012	1.09 (0.95–2.42)	0.096
High UA group	1.18 (0.95–2.71)	0.017	1.02 (0.98–2.68)	0.072

HR, hazard ratio; CI, confidence interval; UA, uric acid; and CVD, cardiovascular disease. Different UA groups, low (<5.0 mg/dL), reference (5–7 mg/dL), and high (>7.0 mg/dL) UA groups; the reference group as reference. The model adjusted for age, sex, dialysis vintage, diabetes mellitus, body mass index, lean tissue index, Geriatric Nutritional Risk Index, hemoglobin, albumin, phosphorus, parathyroid hormone, total cholesterol, triglyceride, serum creatinine, and UA-lowering agents.

**Figure 3:**
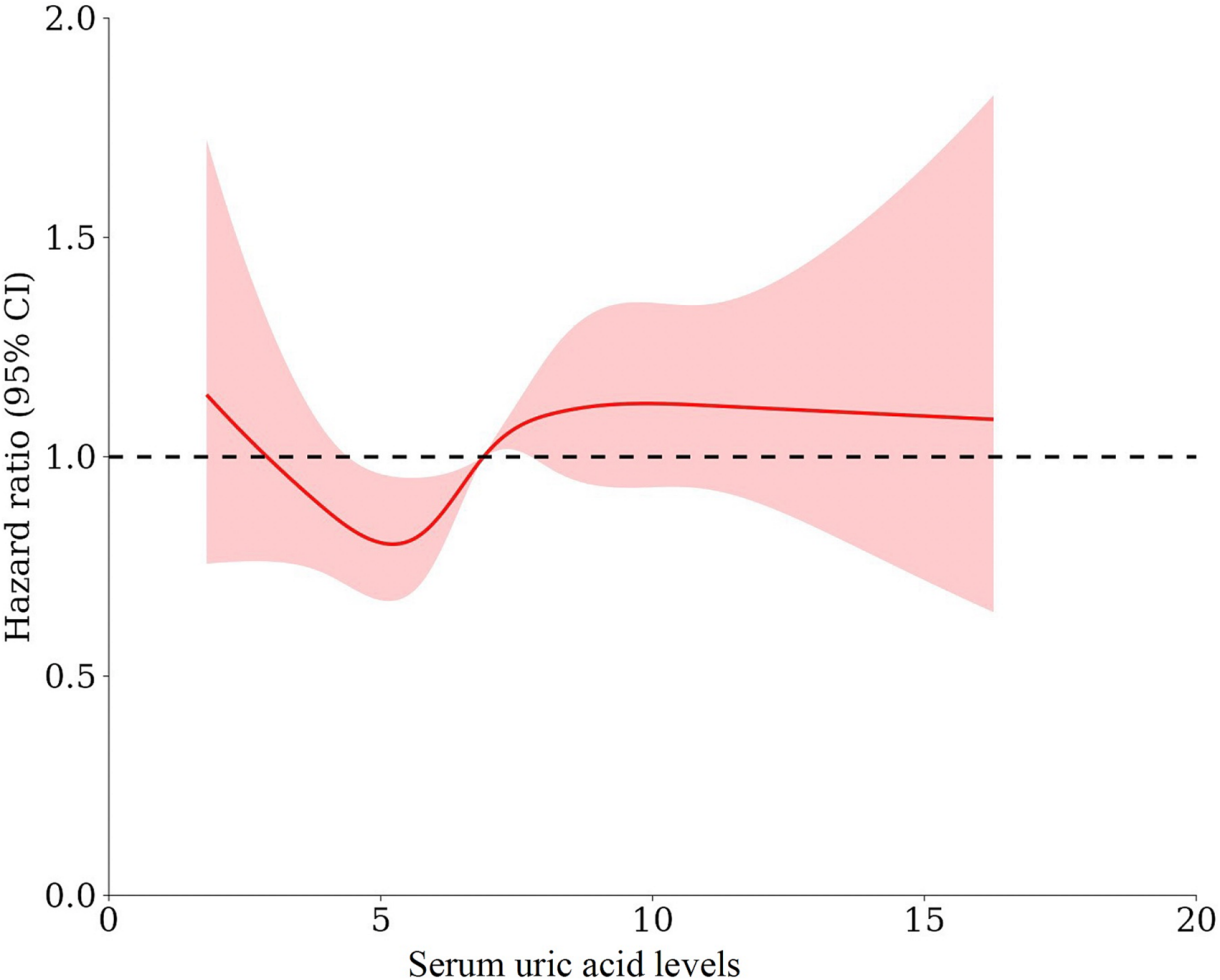
The form of association between the serum UA levels and all-cause mortality using restricted cubic splines A non-linear U-shaped pattern was observed between the UA levels and all-cause mortality after adjusting for age, sex, dialysis vintage, diabetes mellitus, body mass index, lean tissue index, Geriatric Nutritional Risk Index, hemoglobin, albumin, phosphorus, parathyroid hormone, total cholesterol, triglyceride, serum creatinine, and UA-lowering agents (*p* = 0.007), with a survival benefit for the patients with serum UA levels between 3.4 and 6.8 mg/dL. The lowest HR was observed at a serum UA level of 5.2 mg/dL (HR = 0.92 CI 0.86–0.98).

**Figure 4:**
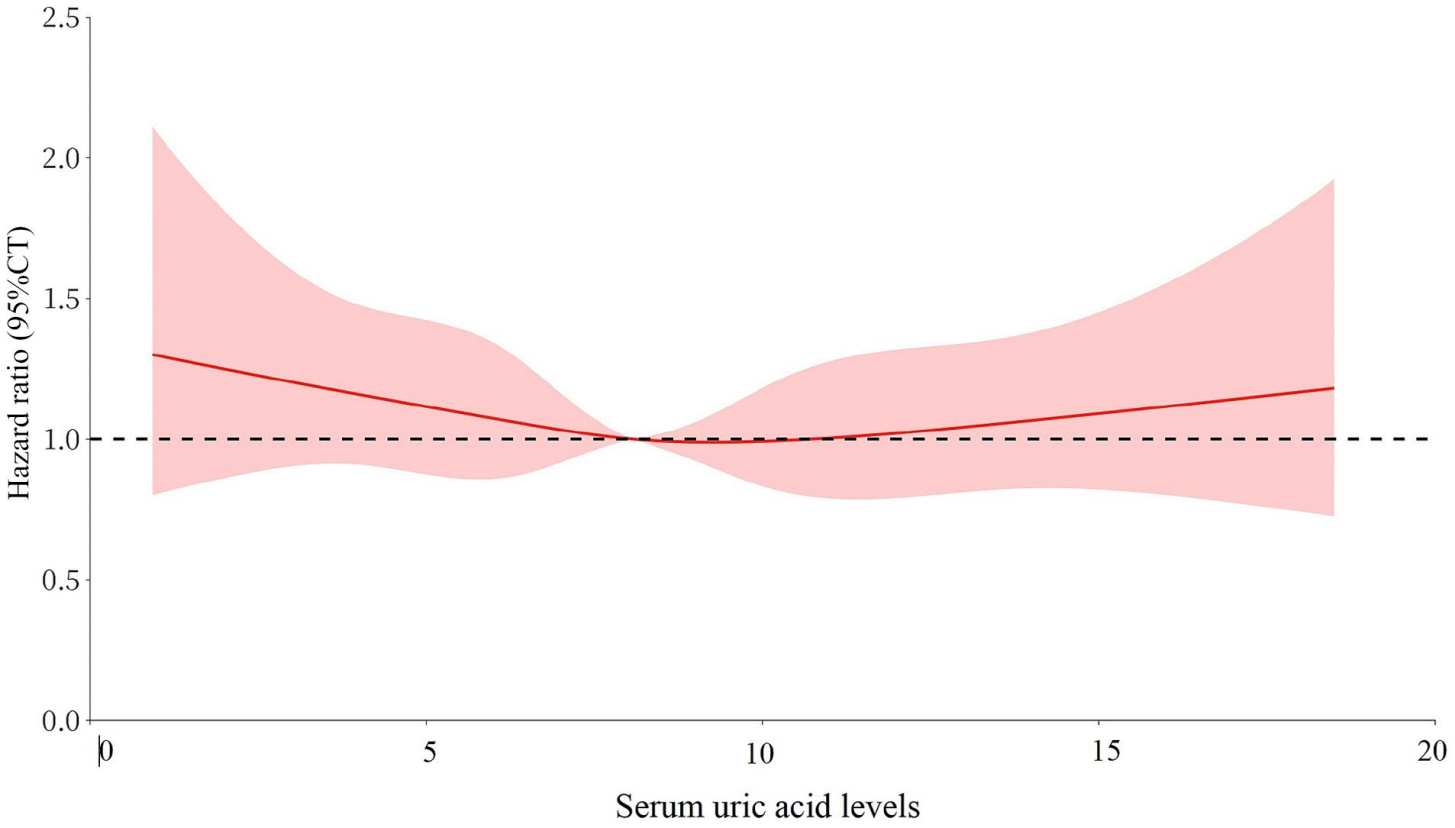
The form of association between the serum UA levels and cardiovascular-related mortality using restricted cubic splines There was a trend toward a higher mortality risk at both lower and higher serum UA levels after adjusting for age, sex, dialysis vintage, diabetes mellitus, body mass index, lean tissue index, Geriatric Nutritional Risk Index, hemoglobin, albumin, phosphorus, parathyroid hormone, total cholesterol, triglyceride, serum creatinine, and UA-lowering agents. However, this association did not reach statistical significance (*p* = 0.12).

These associations were confirmed in the multivariable-adjusted models with restricted cubic splines, where the UA levels were modeled as a continuous variable (Figure 3). We found a non-linear U-shaped pattern between the UA levels and all-cause mortality after multivariable adjustment (*p* = 0.007), with a survival benefit for the patients with serum UA levels between 3.4 and 6.8 mg/dL. The lowest HR was found at a serum UA level of 5.2 mg/dL (HR = 0.92 CI 0.86–0.98); both lower and higher serum UA levels were associated with a greater risk of mortality. For CVD-related mortality (Figure 4), there was a trend toward a greater mortality risk at both lower and higher serum UA levels after full adjustment; however, this association did not reach statistical significance.

**Figure 5:**
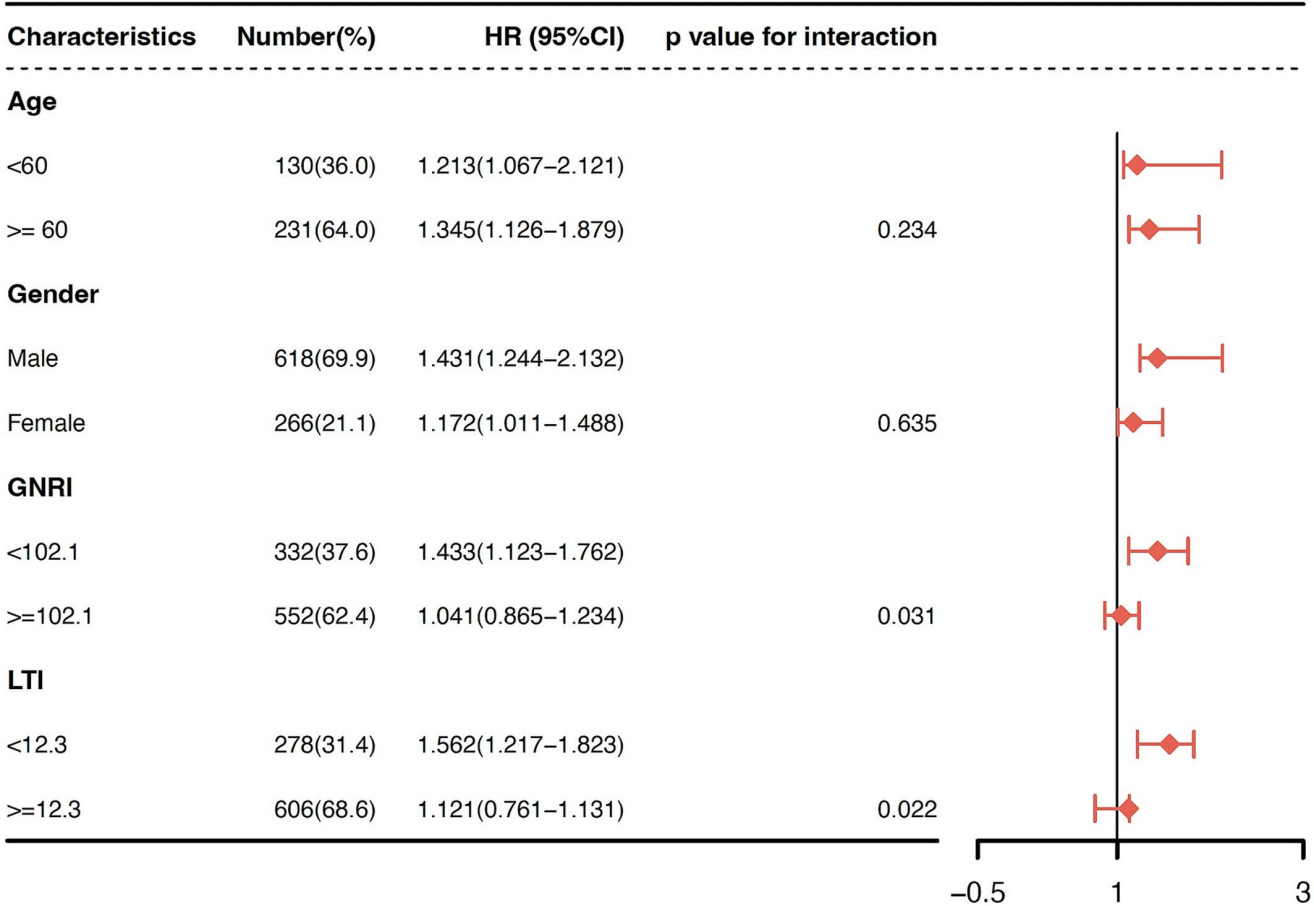
Subgroup analyses for effect modification stratified by age, sex, LTI, and GNRI in the low UA group. The association between the UA levels and all-cause mortality was significantly modified by both LTI (P interaction = 0.022) and GNRI (P interaction = 0.031) in the low UA group. A greater risk of mortality was observed among the patients in the low LTI (<12.3; HR 1.56, 95% CI 1.22–1.82) and low GNRI (<102.1; HR 1.43, 95% CI 1.12–1.76) subgroups, but not among those in the high LTI (≥12.3) and high GNRI (≥102.1) subgroups.

Finally, we analyzed the form of association between serum UA levels and all-cause mortality within the age, sex, LTI, and GNRI subgroups to identify any potential effect modification. Due to the absence of established cut-off values for the LTI and GNRI, we stratified the patients according to the median values of each index. In the multivariable-adjusted model, the association between UA and all-cause mortality was significantly modified by both the LTI (P interaction = 0.022) and GNRI (P interaction = 0.031) in the low UA group (Figure 5). A greater risk of mortality was observed among the patients in the low LTI (<12.3; HR 1.56, 95% CI 1.22–1.82) and low GNRI (<102.1; HR 1.43, 95% CI 1.12–1.76) subgroups, but not among those in the high LTI (≥12.3) and high GNRI (≥102.1) subgroups. The association between UA and all-cause mortality did not change across the age, sex, LTI, and GNRI subgroups in the high UA group. A sensitivity analysis performed to assess the robustness of the association between UA and all-cause mortality, using the mean values of serum UA, revealed similar results.

## Discussion

In this retrospective study of a large cohort of patients who underwent HD, we found that the serum UA levels correlated with the LTI and GNRI scores, which are proxy indicators of nutritional status. Moreover, we found a U-shaped pattern between serum UA levels and all-cause mortality, with a survival benefit for the patients with serum UA levels between 3.4 and 6.8 mg/dL, and the lowest HR was observed at serum UA levels of 5.2 mg/dL. The results of this study indicate that both higher and lower serum UA levels were associated with a higher risk of all-cause mortality among HD patients. Moreover, lower serum UA levels were associated with a higher mortality risk, especially among the patients with low GNRI and LTI scores. However, the serum UA levels did not predict CVD-related mortality in this population. These findings are in contrast with the published literature on the general population, where hyperuricemia has been shown to be associated with an increased risk of gout, hypertension, atherosclerosis, CVD, and mortality (1, 4–11).Our results contribute to the ongoing debate regarding the clinical implications of serum UA changes in patients who undergo HD, as current evidence is conflicting, with reports of direct, inverse, or varying forms of associations (3, 16, 17, 20, 22–25).

The mechanism underlying the inconsistencies between serum UA and all-cause mortality among HD patients may be attributed to the fact that UA has various functional properties. First, UA could be considered an indicator of nutritional status among patients who undergo HD. Consistent with previous studies, we also found that serum UA levels are significantly associated with the nutritional status of patients who undergo HD, as these levels correlate with the LTI and GNRI scores, both of which are useful tools for monitoring nutritional status in HD patients (14–16). Studies have shown that longitudinal changes in serum UA levels seem to reflect changes in nutritional status over time and that these changes are associated with long-term survival in patients who undergo HD (14, 15). Some studies have found that higher UA levels were positively correlated with the normalized protein catabolic rate (14, 23), which is often used as a measure of daily protein intake among HD patients. A high-protein diet can increase protein nitrogen balance, resulting in higher serum albumin levels and increased muscle mass. Therefore, lower serum UA levels have been proposed as a marker for protein-energy wasting in HD patients due to inadequate protein intake, which may partly explain the relationship between lower serum UA levels and higher mortality (14–16, 33). It is noteworthy that in our subgroup analyses, hypouricemic patients with high LTI and GNRI values had a lower mortality risk compared to hypouricemic patients with low LTI and GNRI values. The effect modification of the LTI and GNRI on the association between UA and all-cause mortality leads us to speculate that patients with adequate protein intake and good nutritional status may not have a higher risk of mortality, even if they have low UA levels. Second, oxidative stress is strongly linked with inflammation (34) and poor survival (35) in patients who undergo HD. UA has also been found to have both antioxidant and pro-oxidant properties, depending on its concentrations in the body. It may act as a potent antioxidant at physiological levels or as a pro-oxidant at high, supraphysiological levels (36). On the one hand, UA has been shown to have antioxidant properties *in vitro* (37). In this context, UA has been linked to some of the survival benefits observed in the HD population (15). Therefore, it has been suggested that elevated serum UA levels may be a result of the body’s compensatory mechanism to counteract oxidative damage (12, 22). Conversely, a low serum UA level may reflect inadequate protection against oxidant-mediated stress (13). In addition, given that patients who undergo HD are exposed to various oxidative stresses, the antioxidant properties of slightly elevated UA levels may play a role in achieving better survival outcomes. On the other hand, it is important to note that in certain situations, UA may act a pro-oxidant. Both clinical and experimental studies have provided evidence of the proinflammatory effects of UA in human vascular smooth muscle and endothelial cells, along with a reduction in nitric oxide levels (38–41). This is especially the case when serum UA levels exceed supranormal levels in the blood (36, 42, 43). In addition, overproduction of UA, which is usually associated with metabolic syndrome, may also reflect the underlying inflammatory state, subsequently increasing the risk of mortality (41, 44). Although high UA levels may be associated with all-cause mortality in HD patients, it is important to clarify whether hyperuricemia itself is a risk factor for mortality.

The present study did not find an association between UA and CVD-related mortality. Similarly, a longitudinal cohort study on patients who underwent maintenance HD indicated that a longitudinal change in serum UA levels over time was associated with all-cause mortality but not with cardiovascular mortality or the occurrence of the first cardiovascular event (14). The inconsistent variation in UA levels in relation to cardiovascular mortality may be explained by its different functional effects on cardiovascular outcomes in HD patients. Another reason is that CVD deaths may have been underestimated in our study as a proportion of the patients included died due to infections during the COVID-19 outbreak. In addition, the nutritional status represented using UA levels had a greater impact on mortality during the outbreak.

Our study has several strengths. It was a cohort study that included a large number of patients who underwent HD in a single center, with detailed data collected by the same medical team. This reduced variability in the measurement results between different dialysis centers and allowed for comprehensive statistical adjustments to minimize bias. In addition, the use of penalized smoothing splines in our analysis revealed a smooth U-shaped curve, enabling the evaluation of the risks at extreme serum UA levels. This is also an advantage compared to the majority of the previous studies that used internal cutoffs (such as quartiles of distribution). The nutritional status was assessed using the LTI as an anthropometric parameter and the GNRI as a dietary intake parameter, both of which were validated in Asian patients who underwent HD (45). Our study also has some limitations. First, there is inherent bias in single-center retrospective studies, where associations do not prove causality. Second, residual confounding factors, such as normalized protein catabolic rate, alcohol consumption, medications affecting uric acid, and other unmeasured confounders, cannot be excluded. In addition, we excluded peritoneal dialysis patients from the study, which could have introduced potential bias. Third, we analyzed the serum UA levels only at the index date, which did not account for the changes in the serum UA levels during the observation period. As a retrospective study, the patients’ uric acid measurements were not taken regularly, so we were unable to perform a trajectory analysis. Therefore, we chose to analyze the effect of the mean value of the serum UA levels on mortality during the follow-up period as a sensitivity analysis. It is important to note that, as the majority of previous studies used a single serum UA measurement, we adopted the same design for comparability reasons. Finally, the cause of death was determined based on death certificates and expert consensus, and it was not confirmed through autopsies, which could have introduced bias and inaccuracies. However, despite these limitations, we believe our study contributes to the limited understanding of the role of UA in HD patients.

In conclusion, we found a U-shaped pattern between serum UA levels and all-cause mortality among HD patients. Compared to the normal reference range for UA, both lower and higher levels of UA were associated with a greater risk of mortality. This finding suggested that better nutritional status, rather than high serum UA levels, is likely to improve the long-term survival of patients who undergo HD. These findings highlight the need for simple laboratory tests to estimate serum UA levels to assess disease prognosis and identify the need for nutritional interventions. Future prospective studies are needed to explore the impact of UA and nutritional status on long-term survival in HD patients.

## Data Availability

The original contributions presented in the study are included in the article/supplementary material, further inquiries can be directed to the corresponding authors.
